# Current landscape and future perspectives in preclinical MR and PET imaging of brain metastasis

**DOI:** 10.1093/noajnl/vdab151

**Published:** 2021-10-14

**Authors:** Synnøve Nymark Aasen, Heidi Espedal, Olivier Keunen, Tom Christian Holm Adamsen, Rolf Bjerkvig, Frits Thorsen

**Affiliations:** 1 Department of Biomedicine, University of Bergen, Bergen, Norway; 2 Department of Health and Functioning, Western Norway University of Applied Sciences, Bergen, Norway; 3 The Molecular Imaging Center, Department of Biomedicine, University of Bergen, Bergen, Norway; 4 Mohn Medical Imaging and Visualization Centre, Department of Clinical Medicine, University of Bergen, Bergen, Norway; 5 Translational Radiomics, Department of Oncology, Luxembourg Institute of Health, Luxembourg, Luxembourg; 6 Centre for Nuclear Medicine, Department of Radiology, Haukeland University Hospital, Bergen, Norway; 7 180 °N – Bergen Tracer Development Centre, Department of Radiology, Haukeland University Hospital, Bergen, Norway; 8 Department of Chemistry, University of Bergen, Bergen, Norway; 9 NorLux Neuro-Oncology Laboratory, Department of Oncology, Luxembourg Institute of Health, Luxembourg, Luxembourg; 10 Department of Neurosurgery, Qilu Hospital of Shandong University and Brain Science Research Institute, Shandong University, Key Laboratory of Brain Functional Remodeling, Shandong, Jinan, P.R. China

**Keywords:** brain metastasis, clinical imaging, MRI, PET, preclinical imaging

## Abstract

Brain metastasis (BM) is a major cause of cancer patient morbidity. Clinical magnetic resonance imaging (MRI) and positron emission tomography (PET) represent important resources to assess tumor progression and treatment responses. In preclinical research, anatomical MRI and to some extent functional MRI have frequently been used to assess tumor progression. In contrast, PET has only to a limited extent been used in animal BM research. A considerable culprit is that results from most preclinical studies have shown little impact on the implementation of new treatment strategies in the clinic. This emphasizes the need for the development of robust, high-quality preclinical imaging strategies with potential for clinical translation. This review focuses on advanced preclinical MRI and PET imaging methods for BM, describing their applications in the context of what has been done in the clinic. The strengths and shortcomings of each technology are presented, and recommendations for future directions in the development of the individual imaging modalities are suggested. Finally, we highlight recent developments in quantitative MRI and PET, the use of radiomics and multimodal imaging, and the need for a standardization of imaging technologies and protocols between preclinical centers.

Brain metastases (BM) represent the most frequently found brain malignancies, commonly developed from lung cancer (40–50%), breast cancer (15–25%), or malignant melanoma of the skin (5–20%).^1^ Untreated, the median survival for patients diagnosed with BM is 3–4 months,^2^ while aggressive treatment may increase the median survival to around 15 months.^3^

Clinical and preclinical BM imaging is primarily used for diagnosis, prognosis, and assessment of treatment responses. Predicting clinical effects of novel drug candidates from results obtained in BM animal models is difficult and nine out of ten promising strategies have failed in subsequent clinical trials.^4^ This emphasizes the need for high quality preclinical imaging data that better support decision-making processes for clinical translation.

In this review, we describe and compare the use of MRI and PET imaging technologies used in preclinical and clinical management of BM. In particular, we focus on established and recent developments in MR and PET imaging strategies applied to preclinical BM animal models, identifying their strengths and shortcomings. Based on this we provide recommendations for future developments in preclinical imaging that may facilitate translation into the clinic. Our recommendations are based on an extensive literature search up to February 2021.

## Magnetic Resonance Imaging (MRI)

A summary of the MRI techniques used in clinical and preclinical imaging of BM is provided in Table 1, and an overview of the typical spatial resolution of the different techniques is found in Supplementary Table 1.

**Table 1.** MRI Techniques Relevant for Clinical and Preclinical Imaging of Brain Metastasis

MRI Technique	Clinical Utilization	Clinical References	Preclinical Utilization	Preclinical References
Anatomical MRI	Monitor BM development, define tumor border, study edema	^6,7^	Monitor BM development, define tumor border, study edema	^8–11^
bSSFP imaging	Differentiate between BM and gliomas	^17^	Study BBB integrity, cell tracking, improved BM detection	^18–24^
DSC-MRI	Separate GBM from BM, distinguish pseudo-progression from disease progression	^27,28^	Compare rCBV between BM and normal brain	^29^
DCE-MRI	Distinguish between disease progression and pseudoprogression, tumor differentiation, study therapeutic responses, and changes in permeability	^28,33–35^	Characterize BM vasculature, study treatment effects	^36,37^
ASL	Differentiate primary tumors and BM, diagnosis of BM, assess BM progression	^38,39^	Study CBF in BM	^40^
DWI	Estimate survival after surgery, predict treatment response	^42,43^	Study BM growth, distinguish edema, and CSF from BM	^44,45^
DTI	Differentiate high grade gliomas from BM, distinguish between gliomas and BM, preoperative planning of surgery	^38,47–49^	None	None
Cellular MRI	None	None	Tumor cell tracking, study BBB integrity, blood tumor volume and vascularity, earlier detection of BM	^8,10,15,21,54,56,57^
CEST imaging	Prediction of true volume changes, distinguish between tumor progression and radiation necrosis	^60–62^	Study amide proton levels in BM compared to normal brain	^63^
^1^H-MRS	Differentiate between BM and gliomas	^64,66^	Tumor metabolomics, monitor radiation effects	^44,67^
^31^P-MRS	Differentiate between BM and gliomas, metabolic studies	^68,69^	None	None
BOLD	Tumor vascularization in BM	^70^	Cortical activation in BM compared to normal brain	^71,72^

DSC, dynamic susceptibility contrast; DCE, dynamic contrast enhanced; ASL, arterial spin labelling; DWI, diffusion weighted imaging; DTI, diffusion tensor imaging; CEST, chemical exchange saturation transfer; bSSFP, balanced steady-state free precession; ^1^H-MRS, proton magnetic resonance spectroscopy; ^31^P-MRS, phosphorus magnetic resonance spectroscopy; fMRI, functional magnetic resonance imaging; BOLD, blood oxygenation level-dependent imaging; GBM, glioblastoma multiforme; BM, brain metastasis; rCBV, relative cerebral blood volume; CBF, cerebral blood flow; CSF, cerebrospinal fluid; BBB, blood–brain barrier.

### Anatomical MRI

Anatomical brain MRI represents a standardized technique providing rich information on shape, volume, and integrity of the brain including alterations of the blood–brain barrier (BBB).^5^

Brain regions are separated using T1-weighted images, whereas T1-weighted images following contrast agent (CA) injections are used to visualize tumors having a leaky vasculature. Edematous regions can be delineated in T2 images and separated from CSF by FLAIR images.^6^ Thus, contrast-enhanced T1-weighted MRI and FLAIR represent the most common imaging methods clinically, providing information on tumor size and morphology.^7^

Due to its superior soft tissue contrast, anatomical MRI has been used in preclinical studies to monitor BM development from melanoma,^8,9^ breast,^10^ and lung cancer.^11^ Yet, the technique shows difficulties in differentiating BM from high-grade primary brain tumors or infections,^12^ discriminating pseudoprogression from true tumor progression,^13^ and separating radionecrosis from recurrent tumors.^14^ In the few cases of infiltrative BMs, the tumor border may be underestimated.^15^

Albeit with some limitations, anatomical MRI represents an important method, to evaluate BM progression and treatment efficacy in preclinical and clinical studies.

### Balanced Steady-State Free Precession (bSSFP) Imaging

bSSFP represents a fast anatomical MRI method that uses steady states of magnetization. In general, these sequences are based on a low flip angle gradient echo MRI sequence with a short repetition time, a fast low-angle shot (FLASH) technique.

Clinically, most data on bSSFP has been obtained on cardiac imaging,^16^ and very little has been described on brain tumor imaging. In a previous study, bSSFP was applied for quantitative magnetization transfer analysis (qMT) on patients with gliomas (15 patients), meningiomas (4 patients), and BM (7 patients). The study indicated that qMT may enable a more subtle differentiation between brain tumors, complementing conventional T1 and T2 weighted MRI. However, the authors pointed out the need for a validation in a larger patient cohort.^17^ The technique is sensitive to magnetic field inhomogeneities, making it attractive to image iron containing compounds, such as blood or exogenous particles.

bSSFP has been used in preclinical BM models, to study BBB integrity^18,19^ or track iron-oxide labelled single cells in the mouse brain.^20–24^ In a study on brain metastatic outgrowth of human breast cancer cells, T1 weighted MRI after CA injection was combined with bSSFP to show that radiotherapy did not increase BM permeability. Interestingly, additional lesions not seen by T1 weighted CA MRI, could be detected by bSSFP.^18^

bSSFP provides very good fluid-to-soft tissue contrast, a good signal to noise ratio (SNR), and high spatial resolution, within a short scan time.^25,26^ Next to iron, other sources of field inhomogeneities can however confound the signal and lead to susceptibility artifacts.^23^ Other limitations involve transient oscillation and eddy current effects, which may be solved by special phase-encoding techniques and the use of prescans. Banding artefacts in the regions of magnetic field inhomogeneities may be avoided by short TR and optimal shimming.^26^

To conclude, bSSFP is a suitable technique for the detection of single, prelabelled tumor cells, and for the visualization of brain lesions that are otherwise not seen by regular MRI.

### Perfusion MRI

Three main perfusion techniques exist that provides information on physiological parameters such as blood volume, blood flow, vessel permeability, and vascular supply.

#### Dynamic susceptibility MRI (DSC-MRI).

—In DSC-MRI, a paramagnetic CA is injected, whereupon rapid repeated images are acquired to quantify susceptibility induced signal loss, based on changes in T2 or T2* relaxivity. The technique has been used in the clinic to show that the relative cerebral blood volumes (rCBV) in the peritumoral brain areas were significantly higher in glioblastomas (GBMs) compared to BM, enabling distinction between the two malignancies, likely due to a higher vascular density in GBMs. The sensitivity and specificity in the diagnosis of GBMs were 94.44% and 91.67%, respectively.^27^ It has further been demonstrated that rCBV values may be used to differentiate between pseudoprogression and disease progression in BM following stereotactic radiotherapy, with sensitivity, specificity, and overall diagnostic accuracy of 80%, 86%, and 83%, respectively.^28^

Preclinically, a brain tropic breast cancer cell line was injected intracardially into nude mice. DSC-MRI showed that rCBV values in BM were significantly lower than in the contralateral normal brain, verified by histological analysis of microvascular density.^29^

The method is commonly used for perfusion imaging, as it is easy to implement, has a short duration, and provide information on blood volume, blood flow, and transit time. High-speed acquisitions are needed to obtain a good temporal resolution, limiting spatial resolution, and SNR.^30^

In conclusion, DSC-MRI perfusion studies should be explored further in BM animal models where the aim should be to see if DSC-MRI data from such models reflect results obtained in the clinic.

#### Dynamic contrast enhanced MRI (DCE-MRI).

—In DCE-MRI, T1-weighted images are acquired before, during, and after intravenous injection of a CA. Perfusion parameters are obtained from CA time curves derived from changes in T1 relaxivity within the tissue. DCE-MRI delineates changes in vessel perfusion, permeability, and vascular and extravascular volume fractions.^31^ The method is well suited to assess antiangiogenic effects of drugs at earlier timepoints than DSC-MRI.^32^ Clinically, it has been shown that DCE-MRI derived volume transfer constant (K^trans^) can be used to distinguish pseudoprogression from disease progression, with sensitivity, specificity, and overall diagnostic accuracy of 90%, 68%, and 79%, respectively.^28^ The diagnostic utility of the perfusion parameters K^trans^, extravascular extracellular volume V_e_, and fractional plasma volume V_p_ for differentiating lymphomas from other malignant brain tumors has also been demonstrated.^33^ A pharmacokinetic model free approach to predict therapeutic responses to radiation therapy was also proposed, based on Principal Component Analysis of the Area Under the Curve (AUC) of the DCE signal.^34^ Also, increases in blood-tumor barrier (BTB) permeability were assessed by changes in K^trans^ values in order to determine an optimal time for start of systemic therapy.^35^

**Figure 1.**
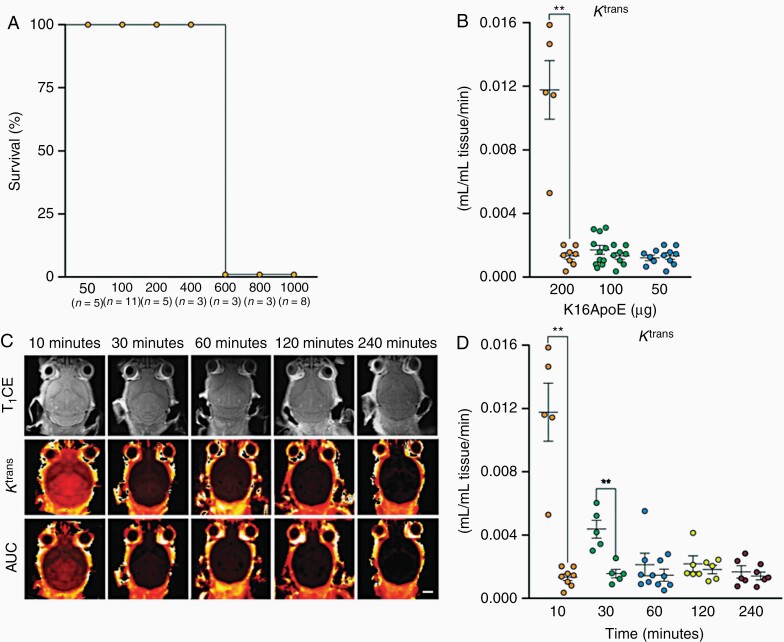
**The synthetic peptide K16ApoE transiently opens the blood–brain barrier and creates a time window for therapy for at least 30 min.** (A) Survival curves after K16ApoE dose escalation experiments (peptide concentrations are given in μg K16ApoE in the X-axis). (B) Scatter plots of the blood-to-tissue transfer constant (K^trans^) demonstrate a dose-dependent reduction of contrast agent transfer from blood to tissue with decreasing peptide concentrations (*n* = 5–10 mice). Mean ± SEM. (C) Representative anatomical contrast enhanced T_1_ weighted MR images (top row) and parametric MR images (K^trans^ and AUC maps; 2 bottom rows) of coronal brain sections of a control mouse. Scale bar, 2.5 mm. (D) The quantified K^trans^ analysis demonstrates leakage of contrast agent from blood to tissue 10 and 30 min after injection of 200 mg of K16ApoE, compared with control animals (*n* = 5–10 mice). Mean ± SEM. K^trans^, transfer constant; T_1_CE, contrast enhanced T_1_ weighted scan; ^**^, *P* < 0.01. Reprinted from Aasen SN et al.^37^ with permission from Mol. Cancer Ther.

Preclinically, quantitative DCE-MRI analysis showed significantly higher K^trans^ values in a breast cancer BM model treated with whole brain radiation therapy, compared to the control group.^36^ Changes in K^trans^ and AUC values were used to demonstrate changes in BBB permeability in a melanoma BM model after combined use of dabrafenib and a peptide that transiently opens the BBB (Figure 1).^37^

At present, the method is not widely implemented preclinically, and there is a need for improved standardization of scan protocols and pharmacokinetic analysis models to compare results between institutions.^32^ Results may also vary based on the type of anesthesia used, animal body temperature, magnetic field strength, and methods for CA delivery.^31^

In conclusion, DCE-MRI is an effective method to study changes in vascular parameters in experimental BM models and in the clinic. Moreover, the method is highly suited to assess the effects of various BBB opening strategies, which represent an important research domain for increased drug delivery to the brain.

#### Arterial spin labelling (ASL).

—ASL measures tissue perfusion using magnetically labelled blood water protons, conveniently dispensing the need for exogenous CAs.

In the clinic, ASL combined with diffusion tensor imaging (DTI) has been shown to differentiate primary brain tumors from BM with a diagnostic accuracy of 97%, due to higher tumor blood flow of both tumoral and peritumoral parts in GBMs, compared to BM.^38^ Also, BM recurrence after stereotactic radiosurgery could be determined with a sensitivity of 87% and specificity of 100%.^39^

Preclinically, metastatic murine mammary carcinoma cells were injected into the striatum of mice. A multiphase pseudo continuous ASL technique was then used to study cerebral blood flow (CBF). The study showed a reduced CBF in the BMs after 35 days, despite a higher blood vessel density. Also, the tumor core exhibited a reduced blood flow compared to the tumor rim.^40^

ASL provides absolute quantitative measurements of blood flow, and is relatively easy to implement.^40^ The technique benefits from the high magnetic field strengths commonly used preclinically, providing enhanced signal differences.^32^ Major concerns related to the ASL technique involves the loss of signal due to susceptibility artefacts, motion artefacts and low SNR.^13^ Preclinically, interspecies variations in for instance head geometry and air cavities in the rodent head may also lead to inhomogeneous magnetic fields, resulting in variations in CBF results.^41^

In short, the ASL method needs further development to fully exploit its potential for assessment of tissue perfusion in preclinical BM models. Moreover, ASL results from such models need to be validated in clinical studies.

### Diffusion Weighted Imaging (DWI)

DWI is an imaging technique that produces signals based on diffusion of water molecules, without using an exogenous CA. By measuring the displacement of water molecules diffusing across the tissue per time unit, apparent diffusion coefficient (ADC) maps can be calculated, providing functional information not available from structural MRI.

Clinically, it has been shown that ADC, combined with clinical scoring systems, represents a simple method that improves survival estimation in patients having BM surgery.^42^ Altered ADC values combined with information on tumor volumes of BM in patients undergoing radiation therapy also represent a reliable method for the prediction of treatment responses.^43^

**Figure 2.**
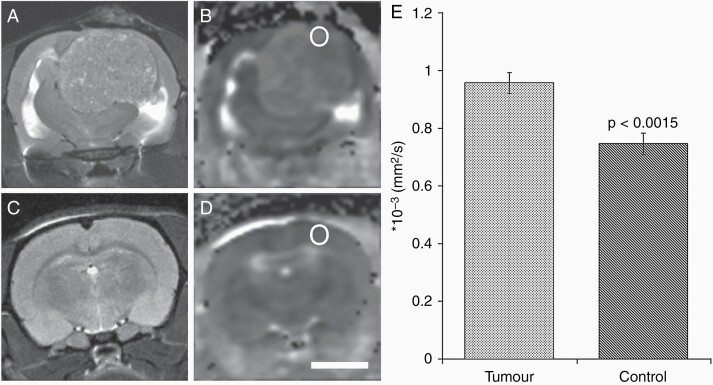
**Magnetic resonance (MR) diffusion properties in experimental brain metastases.** (**a**) Representative T2 weighted MR image of a rat brain harboring a colon metastasis. (**b**) The corresponding apparent diffusion coefficient (ADC) map of the tumor shown in (**a**). (**c**) T2 weighted MR image of a control animal, with the corresponding ADC map shown in (**d**). The cortical ADC values were investigated by drawing a circular region of interest on the ADC images (seen on **b** and **d**). Scale bar: 5 mm. (**e**) Comparison of the mean ADC values from six tumor animals and four control animals. There was a 28% increase in the ADC in tumor animals, as compared with control animals. Reprinted from Wang J et al.^45^ with permission from Neuropathol. Appl. Neurobiol.

Preclinically, ADC maps have been used to evaluate BM growth from human mammary carcinoma cells inoculated into the internal carotid artery of nude mice.^44^ BM tissue from a colon cancer patient was implanted directly into the brains of immunodeficient rats. Increased intratumoral edema was observed through increased ADC values, compared to normal tissue (Figure 2).^45^

DWI is relatively easy to perform. However, variations in technical parameters such as field strength, shimming, choice of b-values, and cut-off values for different DWI variables may lead to substantial disparities in clinical and preclinical results.^46^

To conclude, DWI is a promising preclinical method to evaluate BM growth and development of edema. Standardization of the technique between centers, both in a preclinical and clinical context is encouraged to fully exploit its potential.

### Diffusion Tensor Imaging (DTI)

DTI is used to measure restricted tissue diffusion of water in multiple directions. In the clinic, DTI has been employed to differentiate high grade gliomas from BM. Directional diffusion indices have been used to calculate several anisotropic values. Metastatic tumors are usually well circumscribed, pushing nerve fibers away instead of infiltrating them. As a consequence, anisotropy variables tend to be lower in BM compared to gliomas.^47^ Others have combined ASL and DTI to distinguish GBMs from BM. It has been shown that a combination of tumor blood flow (TBF) and fractional anisotropy (FA) values in the tumoral and peritumoral areas could be used to separate gliomas from BM, with a sensitivity of 86.7% and 86.7%, and a specificity of 95.2% and 90.5% (tumoral vs. peritumoral part).^38^ DTI combined with DWI has also been shown to discriminate solitary BM from high grade gliomas with 88% sensitivity and 90% specificity, by combining parameters such as AUC, mean diffusivity, and fractional anisotropy.^48^ Further, DTI may provide crucial information in preoperative planning before glioma and BM surgery, that is to plan the surgical route and avoid white matter tracts.^49^

Preclinically, DTI has not been used in BM models, although it represents a promising technique for mapping complex microstructures in the rodent brain. However, long scan times are needed to achieve a sufficient SNR and a high isotropic resolution.^50^ Also, the method is sensitive to eddy currents and motion-induced artefacts.^47^

In conclusion, DTI-MRI can be used to differentiate high grade gliomas from BM. DTI-MRI has an unused potential in BM preclinical studies where in particular protocols for reducing scan times should be developed.

### Cellular MRI Using Iron Oxide Nanoparticles as CAs

Cellular MRI may be defined as noninvasive imaging of targeted cells and cellular processes in living organisms. SPIONs shorten T2 and T2* relaxation rates by disrupting local magnetic fields, providing improved contrast in comparison to paramagnetic agents, even at micromolar iron concentrations.^51^ The role of superparamagnetic iron oxide nanoparticles (SPIONs) in MRI diagnosis of BM patients has not been studied to date.

Preclinically, the cells of interest can be labelled with SPIONs prior to MRI, in order to separate them from nearby tissues,^52^ and to provide early tumor detection.^53^ In mice, single human melanoma cells prelabelled with SPION were identified by MRI 24 h after intracardial injections of human melanoma BM cell lines into mice. This led to direct estimation of the ratio between the number of BM cells entering the brain and the number of metastases formed.^8^ Further, SPIONs have been used to compare the efficacy of T2* weighted MRI and phase contrast MRI to detect brain metastatic spread of prelabelled breast cancer cells injected intracardially into rats.^54^

Ultrasmall superparamagnetic nanoparticles (USPIONs) have been used preclinically to assess the brain tumor vasculature, as they remain in the blood system for extended time periods, and may thus represent a suitable tool for evaluating changes in tumor blood volume.^55^ MRI using Magnevist and USPIONs as CA has been performed on mice harboring BM breast cancer cells where the spatial distribution of tumor vascular parameters, water diffusion, and invasion were assessed. The study showed that dual contrast MRI and evaluation of tissue diffusion properties enabled assessment of brain tumor heterogeneity and that this may be used to develop more informative predictive biomarkers.^15^

Targeted MRI based on microparticles of iron oxide (MPIOs) has been used for visualization of the endothelial vascular cell adhesion molecule-1 (VCAM-1) in the mouse brain, where it was shown that VCAM-1 expression, detected by MRI, increased significantly with tumor progression. Further, VCAM-1 targeted MRI detected micrometastases before gadolinium contrast enhancement.^10,56^ Tumor cell dormancy has also been studied using MPIO labelled human breast cancer cells inoculated into mice. Only 1.5% of the cells reaching the brain were able to establish BMs, whereas a large subset survived in a dormant state as nondividing, single cells.^21^ In another study, MPIO was bound to endothelial activated leukocyte cell adhesion molecule (ALCAM) and injected intravenously into three different BM animal models. ALCAM-MPIO induced hypointensities were seen on T2* weighted MRI in all models, while post gadolinium MRI showed that the BBB was intact.^57^

Preclinical experiments using iron-based cellular MRI have provided valuable insight into many BM processes. Yet, it is likely that some of the findings may be model specific, and a verification across several models is warranted. At present, there is one ongoing clinical Phase II clinical trial (NCT03325166) where ferumoxytol-based CA and gadolinium-based CA are being used in perfusion imaging to measure treatment efficacy in BM nonsmall cell lung cancer (NSCLC) patients.

In short, cellular MRI is an excellent method to study single tumor cells and biological features of BM growth and development preclinically. In the clinic, the techniques have the potential to provide an earlier BM diagnosis.

### Chemical Exchange Saturation Transfer (CEST) Imaging

CEST is an MRI contrast method that can be used to study the tumor microenvironment through detection of mobile proteins and peptides, which are first saturated with a specific radiofrequency pulse, before they transfer their magnetization to unbound water. This results in a reduced MR image signal during acquisition that is proportional to the concentration of the proteins and peptides. By varying the radiofrequencies, a spectrum is generated.^58^ By this method, metabolites can be measured at very low tissue concentrations.^59^

Clinically, CEST imaging has been shown to differentiate gliomas from lung cancer BM by increased magnetization transfer ratio (MTR) values in gliomas compared to BM.^60^ By CEST, the broad magnetization transfer effects as well as the relayed nuclear Overhauser effect (NOE) from aliphatic groups have been assessed. It was found that the NOE peak amplitude could predict volume changes one month after stereotactic radiosurgery (SRS).^61^ Also, it has been shown that NOE may be used to differentiate tumor progression from radiation necrosis following SRS treatment.^62^

Preclinically, human breast cancer cells have been injected directly into the brains of mice. Amide proton transfer (APT) CEST imaging showed a higher APT signal from tumors than normal brain, which correlated with increased cell proliferation, assessed by immunohistochemistry. Also, an increased APT signal was found in tumor areas with elevated bleeding.^63^

The chemical exchange rate in CEST is sensitive to changes in molecular interactions and metabolite concentrations and may thus be suitable for noninvasive measurements of pH.^59^ The contrast is limited by its sensitivity to field inhomogeneities at higher field strengths, especially in the B0 and B1 fields. Consequently, post processing using correction algorithms is needed to compensate for field inhomogeneity-induced artefacts. Also, T1 and T2 tissue relaxation effects might confound the APT CEST signal.^63^

In conclusion, CEST imaging has a potential for studying the tissue microenvironment in preclinical BM models but requires mathematical methods to reduce artifacts.

### Magnetic Resonance Spectroscopy (MRS)

MRS provides metabolomic information about the chemical composition of tissues where it gives information on relative amounts as well as absolute metabolite concentrations.^13^

In a clinical study, GBM and BM lesions were first identified by T1, T2 and T2* MRI, diffusion weighted axial images, and post contrast 3D fast spoiled with CA administration. Single-voxel MRS of the tumors were then acquired. Four lipid peaks (Lip) and 5 macromolecule (MM) peaks were analyzed quantitatively, showing that a combination of four of these components (MM14, Lip13a, Lip13b, and MM12) could be used to discriminate GMB from BM with high specificity and sensitivity.^64^

In chemical shift imaging (CSI), MRS spectra from multiple voxels can be overlaid onto MR images.^65^ In a patient study, CSI was used to differentiate between low grade gliomas (LGG), high grade gliomas (HGG), and BM. Ratios of metabolites were calculated and it was shown that the ratios lipid/choline and myo-inositol/choline combined showed 100% sensitivity and specificity for HGG vs LGG and LGG vs BM.^66^

Preclinically, following injections of breast cancer cells into the left cardiac ventricle of mice, the effects of whole brain radiation therapy were monitored by MRS and MRI. More normalized levels of creatine, N-acetyl aspartate, and lactate were seen, compared to untreated controls.^67^

In a breast cancer BM model, longitudinal MRS showed that N-acetyl aspartate was the first marker of metastatic growth appearing, while choline increased, and creatine levels declined. Also, mobile lipids were observed after a certain period of growth. These results suggest that MRS may be a valuable tool for assessing different tumor progression states and also to monitor treatment efficacy.^44^

Phosphorus (^31^P)-MRS is used to examine high-energy phosphorus metabolism in tissues.^65^^31^P-MRS has been used in the clinic to differentiate the energy status of various primary and secondary brain tumors. Results indicate that inorganic phosphatases and phosphodiesterase represent suitable biomarkers for discriminating gliomas from BM.^68^ In another clinical study,^31^P-MRS showed elevated metabolic changes in contrast enhancing BMs and surrounding areas of edema compared to the normal brain.^69^^31^P-MRS has not been described in BM preclinical studies.

It may be challenging to obtain comparable MRS results between institutions, due to lack of standardized data acquisition, data analysis and reporting, placement and size of the MRS voxels,^64^ and sensitivity to motion artifacts and water suppression.^13^ Furthermore, MRS studies on BM have been performed on relatively small patient groups, which currently limits their clinical value.

The use of ^31^P-MRS is hampered by low sensitivity of ^31^P nuclei and low concentrations of phosphate metabolites, necessitating long acquisition times to improve SNR.^65^ Low phosphatase metabolite concentrations would also limit the usefulness of ^31^P-MRS in preclinical studies.

In conclusion, ^1^H-MRS is suitable for preclinical imaging, but requires standardisation.^31^P MRS is currently not sensitive enough.

### Blood Oxygenation Level-Dependent (BOLD) Imaging

BOLD imaging assesses the magnetic properties of hemoglobin, which varies depending on its oxygenation state. BOLD imaging was initially developed for functional MRI (fMRI), assuming that brain activation is linked to an increase in blood flow in areas with increased neuronal activity.^32^ In a clinical study, patients with gliomas and BM were examined by BOLD imaging to elucidate the impact of tumor vascularization on the BOLD signal while performing a finger-tapping task. A significant signal reduction within the tumors, which correlated with total intratumoral blood volume, was found.^70^

Preclinically, very few studies on BM have been done. Rats were injected with rat mammary metastatic cells directly into the thalamus. Cortical BOLD activation areas in response to whisker pad stimulation were significantly reduced in the hemisphere that correlated with thalamic BM tumor burden.^71^ In another study, the influence of STAT3-mediated astrocyte reactivity on neurovascular function in BM rat models was investigated. Multimodal MRI (T1 weighted, T2 weighted, BOLD) showed that a selective targeting of STAT-3 by the drug WP1066 resulted in an improved cerebrovascular function.^72^

fMRI has been a success in brain activation studies, however, it has been difficult to establish the BOLD technique for brain tumors.^32^ Tissue BOLD effects are relatively small (<5%) and require advanced statistics for reliable quantification. Spatial and temporal resolution is typically low. The technique is affected by body temperature, with lowered temperatures causing reduced blood oxygenation and thus decreased signals, and by susceptibility artifacts.^32^

To conclude, the biological questions that may benefit from BOLD imaging of BM animal models are not clear as of today.

## Concluding Remark on MRI

The preclinical studies discussed in this review have mostly focused on multimodal MR imaging. Based on current knowledge, multimodal MRI may be superior compared to single MRI techniques, in particular when addressing important questions related to tumor diagnosis, delineation, progression, and treatment responses. Based on the high number of MRI techniques available, it should be emphasized that a careful planning is needed in order to implement the right technique that will address the right biological questions.

## Positron Emission Tomography (PET)

PET is a functional imaging technology, where compounds labelled with positron emitting radioisotopes are used as molecular probes to image and measure biochemical processes in living tissue.^73^ A summary of the PET tracers used in clinical and preclinical imaging of BM is found in Table 2.

**Table 2.** PET Tracers Relevant for Clinical and Preclinical Imaging of Brain Metastasis

PET Tracer	Clinical Utilization	Clinical References	Preclinical Utilization	Preclinical References
[^18^F]FDG	Discrimination between BM and GBM, distinguish between BM relapse and radiation induced changes	^76–78^	Drug accumulation, study of LDHA expression in BM	^79,80^
[^11^C]MET	Differentiate between recurrent BM and radiation necrosis, delineate BM	^86–88^	None	None
[^18^F]FET	Characterization of FET uptake, differentiate residual or recurrent BM from treatment-related changes and pseudoprogression	^89,92^	None	None
[^18^F]FDOPA	Distinguish between tumor progression or recurrence and radiation necrosis	^84,93^	None	None
[^11^C]AMT	Differentiate BM from GBM	^95^	None	None
[^18^F]FLT	Response assessment	^96,97^	Detect tumor activity and proliferation, study of LDHA expression in BM	^9^
Na[^18^F]F	Detection of BM, study response after chemotherapy	^99^	None	None
[^18^F]-Fluorocholine	Detection of BM, distinguish between BM, gliomas and benign brain tumors	^100^	None	None
^82^Rb	Detection of BM	^101^	None	None
[^68^Ga]Ga-PSMA-11	Detection of BM	^102^	None	None
[^18^F]PSMA-1007	Detection of BM	^103^	None	None
[^18^F]FMISO	Detection of intra-tumoural necrosis	^104^	None	None

PET, positron emission tomography; BM, brain metastasis; GBM, glioblastoma multiforme; BBB, blood–brain barrier; LDHA, lactate dehydrogenase A; [^18^F]FDG, 2-[^18^F]-fluoro-2-deoxy-D-glucose; [^11^C]MET, L-[*methyl*-^11^C]-methionine; [^18^F]FET, *O*-(2-[^18^F]-fluoroethyl-L-tyrosine; [^18^F]FDOPA, L-3,4-Dihydroxy-6-[^18^F]fluorophenylalanine; [^11^C]AMT, α-[^11^C]-methyl-L-tryptophan; [^18^F]FLT, 3′-deoxy-3′-[^18^F]-fluorothymidine; Na[^18^F]F, sodium [^18^F]fluoride; PSMA, prostate-specific membrane antigen; [^18^F]FMISO, [^18^F]fluoromisonidazole.

### 2-[^18^F]-fluoro-2-deoxy-D-glucose (FDG) PET

[^18^F]FDG PET represents the most commonly used technique,^74^ since increased glucose metabolism is seen in most cancers. [^18^F]FDG is taken up by cell membrane glucose transporters (GLUT) and phosphorylated through intracellular hexokinase activity.^75^

Clinically, it has been recommended to combine [^18^F]FDG PET with MRI structural information to distinguish BM relapse from radiation induced changes after radiation treatment,^76^ and in a study of 117 post radiotherapy patients, recurrent BM could be separated from radiation necrosis with a sensitivity of 96% and specificity of 77%.^77^ In a study of 44 lesions treated with SRS, combined use of [^18^F]FDG PET and MRI showed that tumor growth could be separated from radiation necrosis with 86% sensitivity.^78^ It has been suggested that [^18^F]FDG PET should not be performed less than 6 weeks after completion of radiation treatment, as this would enhance tumor-to-normal-brain uptake ratio, while necrotic tissue would exhibit [^18^F]FDG levels more comparable to normal brain tissue.^76^

Preclinically, [^18^F]FDG PET has been used to study accumulation of lapatinib-loaded human serum albumin nanoparticles (LHNPs) in a syngeneic 4T1 BM mouse model. Tissue distribution following intravenous administration revealed that LHNPs achieved increased delivery to the metastatic brain, which significantly extended the median survival time.^79^ The relevance of the glycolytic protein lactate dehydrogenase A (LDHA) during BM progression was studied in an animal model of melanoma BM. LDHA knockdown did not affect BM burden or animal survival, as assessed by MRI and [^18^F]FDG PET.^80^

However, [^18^F]FDG PET has several limitations related to brain applications. There is a high physiologic glucose metabolism in the normal brain, leading to low contrast between tumor and brain tissue.^81^ Second, cerebral inflammatory processes may lead to an elevated [^18^F]FDG uptake, limiting diagnostic performance.^82^ Also, due to variations in methodologies, the reported clinical performance of [^18^F]FDG PET has varied considerably.^83^

In conclusion, the benefit of using [^18^F]FDG PET preclinically remains uncertain.

### Amino Acid PET

Radiolabeled amino acids have been used for decades in neuro-oncology.^83^ Neutral amino acids are transported into the brain by the L-type amino acid transport system (LAT),^84^ allowing for early detection of BM where the BBB is intact. Radiolabeled amino acids are particularly interesting, based on an increased uptake in tumors compared to normal brain tissue.^74^ Also, they may be more accurate in distinguishing tumor progression from radiation-induced changes in BM patients.^83^

#### L-[methyl-^11^C]-methionine ([^11^C]MET) PET.

—Methionine has mainly two metabolic functions, protein synthesis and the conversion to S-adenosylmethionine. Tumor cells often have increased protein synthesis, transmethylation, and transsulphuration, resulting in an increased uptake of the [^11^C]MET tracer.^85^

Clinically, [^11^C]MET PET has been used to differentiate recurrent BM from radiation necrosis.^86^ In one study, [^11^C]MET PET was performed on patients with BM or glioma previously treated with radiotherapy, and compared to pathological diagnosis (tumor resection or biopsy). [^11^C]MET uptake was significantly higher in brain lesions compared to areas with radiation necrosis. No difference in [^11^C]MET uptake was found between gliomas and BM.^87^ [^11^C]MET was more accurate in delineating the extent of brain tumors than [^18^F]FDG.^88^

Preclinically, [^11^C]MET PET studies on animal BM models have not been published so far. A major shortcoming is the short half-life of ^11^C (20 min), necessitating an onsite cyclotron for [^11^C]MET production.^89^ [^11^C]MET may also be taken up by inflammatory lesions,^90^ which may be a disadvantage.

In conclusion, [^11^C]MET PET is suitable for use in preclinical BM models, and should be explored further. The short half-life necessitates on-site tracer production.

#### O-(2-[^18^F]-fluoroethyl-L-tyrosine ([^18^F]FET) PET.

—FET is a fluorinated tyrosine analogue that is not metabolized or incorporated into proteins.^89^ [^18^F]FET imaging shows lower uptake in inflammatory cells compared to [^18^F]FDG and [^11^C]MET and is more specific in differentiating tumor tissue from inflammation than [^11^C]MET.^91^

In a clinical study, BM patients received [^18^F]FET PET and MRI prior to surgery. [^18^F]FET PET was positive in all patients. Only a partial overlap of areas defined as tumor by MRI and [^18^F]FET PET was seen, indicating that the use of only one of these imaging modalities could underestimate tumor size.^92^ Also, a combined [^11^C]MET and [^18^F]FET uptake study was performed on patients with gliomas or BM previously treated with a combination of surgery, radiotherapy, and SRS. Both [^11^C]MET and [^18^F]FET provided similar contrast in gliomas and BM, and delineated tumor tissue outside the MRI changes. The study concluded that [^18^F]FET PET could be used to differentiate residual or recurrent tumors from treatment-related changes and pseudoprogression.^89^

**Figure 3.**
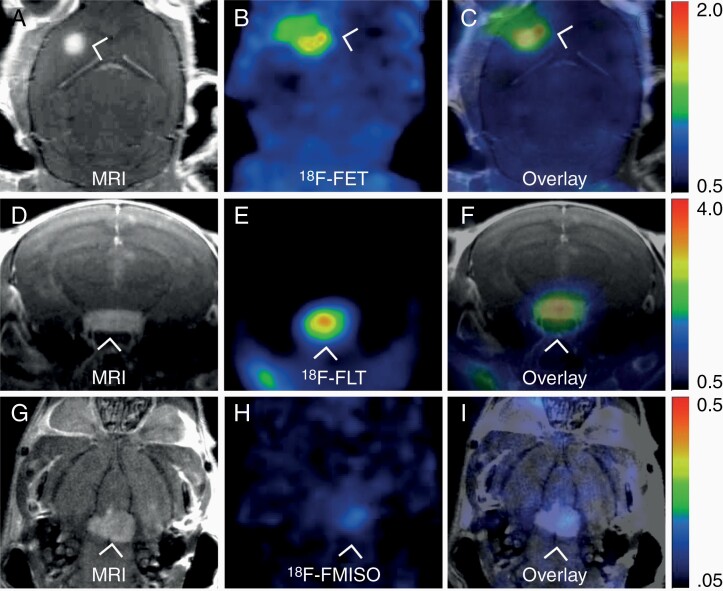
**Multimodal imaging of melanoma brain metastases in mice by contrast-enhanced T1 weighted MRI and PET imaging.** The MR images were acquired on a 7T Pharmascan 70/16 small animal MR (Bruker Biospin MRI GmbH, Ettlingen, Germany) and the PET images were acquired on a nanoscan PET-CT (Mediso Medical Imaging Systems, Budapest, Hungary). (**A**) T1 weighted MR image (coronal orientation) after gadolinium contrast injection, showing hyperintense signal from a brain metastasis of 1.9 mm^3^ (largest diameter 1.4 mm). (**B**) ^18^F-FET PET tracer uptake corresponding to the location of the T1-signal with a SUV_max_ of 1.8. (tumor-brain ratio 2.6). The FET-PET images were acquired 45 min post i.v injection (dose 11.4 MBq). (**C**) Overlay of the MR and ^18^F-FET PET images, showing colocalization of signals from both modalities. (**D**) T1 weighted MR image (axial orientation) after gadolinium contrast injection showing hyperintense signal from a large brain metastasis of 8.7 mm^3^ (largest diameter 3.4 mm). (**E**) ^18^F-FLT PET tracer uptake corresponding to the location of the tumor seen on MRI with a SUV_max_ of 3.8. (tumor-brain ratio 14.6). The FLT-PET images were acquired 30 min after intravenous injection of the tracer (dose 8.1 MBq). (**F**) Overlay of MR and ^18^F-FLT PET images, showing colocalization of signals from both modalities. (**G**) T1 weighted MR image (coronal orientation) after gadolinium contrast injection, showing a hyperintense signal from a 5.2 mm^3^ brain metastasis (largest diameter 3.9 mm). (**H**) ^18^F-FMISO tracer uptake corresponding to the location of the T1-signal shows weak FMISO uptake with a SUV_max_ of 0.3 (tumor-brain ratio 3.3). FMISO images were acquired 90 min after i.v injection (dose 11.0 MBq). (**I**) Overlay of MR and^18^F-FMISO PET images, showing colocalization of signals from both modalities.

Preclinically, research using [^18^F]FET PET in BM animal models has not been published to date. In an unpublished work, we injected human melanoma BM cells into the left cardiac ventricle of nod/scid mice. High intensity signal areas from the PET examinations clearly overlapped with BM detected by MRI, yet [^18^F]FET images indicated tumor outside the area detected by MRI, indicating an intact BBB in that area (Figure 3A–C). The results demonstrate the feasibility of using [^18^F]FET in preclinical BM models, with the ability of detecting tumor areas not visualized by anatomical MRI.

[^18^F]FET has a longer half-life than [^11^C]MET, which is more practical clinically. The simplicity of [^18^F]FET kinetics, with little or no metabolism, is also more convenient for tracer kinetic analysis.^91^

To conclude, [^18^F]FET PET has an unused potential in animal BM imaging, and could be particularly useful in detecting true tumor volumes.

#### L-3,4-dihydroxy-6-[^18^F]fluorophenylalanine ([^18^F]FDOPA) PET.

—[^18^F]FDOPA is the precursor of dopamine, which plays an important role in cerebral coordination of motoric movements.^88^

Clinically, [^18^F]FDOPA PET has been combined with high resolution MRI to distinguish tumor progression or recurrence from radiation necrosis in BM patients.^93^ LAT1 expression levels and [^18^F]FDOPA uptake correlate significantly, which may explain the specific uptake observed in BM patients.^84^ No preclinical studies have yet been performed using this radiotracer.

In general, delineation of tumor margins appears to be comparable when using [^11^C]MET, [^18^F]FET, or [^18^F]FDOPA. A disadvantage with [^18^F]FDOPA, is that physiological uptake in the corpus striatum may prevent a clear definition of tumor margins extending into the basal ganglia.^94^

In conclusion, [^18^F]FDOPA PET has an unused potential in animal BM imaging, especially in models with high LAT1 expression levels.

#### α-[^11^C]-methyl-L-tryptophan ([^11^C]AMT) PET.

—[^11^C]AMT was developed to measure brain serotonin synthesis. Although the literature to date is scarce, [^11^C]AMT PET has also been employed for brain tumor imaging.^91^ In a clinical study, the accuracy of [^11^C]AMT PET in differentiating newly diagnosed GBM from BM was evaluated in patients with suspected brain malignancies. The results indicate that tryptophan accumulation by PET is able to improve pretreatment differentiation.^95^ [^11^C]AMT PET has not been applied to BM models to date.

[^11^C]AMT tumor uptake is comparable to other amino acid tracers and considerably higher than FDG. Due to its short half-life production has to be done locally.^89^

In short, [^11^C]AMT shows similar uptake as other amino acid tracers, and should be explored further in a preclinical context.

### Other PET Tracers

3′-deoxy-3′-[^18^F]-fluorothymidine ([^18^F]FLT) is a structural analogue of the DNA constituent thymidine used to assess cell proliferation. A few clinical studies have used [^18^F]FLT PET to monitor BM growth.^96^ A response assessment was done on patients with BM from breast cancer, before and after treatment with a drug conjugate of paclitaxel and Angiopep-2 (ANG1005). The study reported a moderately strong association between [^18^F]FLT PET and MRI.^97^

In our experience, [^18^F]FLT may be a suitable tracer for detecting tumor activity and proliferation (Figure 3D–F). Preclinical [^18^F]FLT PET was performed after intracardial injections of tumor cells in a well-established melanoma BM model. An accumulation of [^18^F]FLT in BMs was detected four weeks after tumor cell injections.^9^

The [^18^F]FLT tumor uptake depends on thymidine kinase 1 (TK_1_) activity, including therapy-induced activation of the salvage pathway and expression of nucleoside transporters. Therefore, drugs that arrest in the S-phase of the cell cycle may increase [^18^F]FLT uptake. Also, agents that block the endogenous pathway may lead to overactivity of the salvage pathway and also increase the tumor [^18^F]FLT uptake.^98^

Several other radiotracers have been used in clinical assessments of BM, such as sodium [^18^F]fluoride (Na[^18^F]F) to detect BM and study tumor responses after chemotherapy,^99^ [^18^F]-fluorocholine to detect BM and distinguish them from other brain tumors,^100^ rubidium (^82^Rb),^101^ [^68^Ga]Ga-PSMA-11,^102^ and [^18^F]-PSMA-1007^103^ to detect BM, and [^18^F]fluoromisonidazole ([^18^F]FMISO) to study intra-tumoral hypoxia.^104^

None of these PET tracers have so far been described in BM preclinical studies. We recently performed multimodal imaging of a melanoma BM model, using [^18^F]FMISO and T1 weighted MRI after CA injections (Figure 3G–I). Colocalized images showed central hypoxia in single BMs, indicating the efficacy also preclinically to assess tumor development and treatment responses (Figure 3I).

In conclusion, [^18^F]FLT is an interesting candidate for preclinical work. Care must be taken when using therapeutic drugs affecting cell cycle and endogenous cell signalling pathways.

## Current Landscape and Future Perspectives

BM treatment has become more personalized, due to continuous evolvements in diagnostic performance and treatment strategies, which has to some extent improved patient prognosis and quality of life.^7^ Provided a disrupted BBB, most BM can be visualized using anatomical MRI after injecting CA.^83^ If the BBB is intact within the tumors, contrast enhanced MRI techniques will fail to detect them, potentially influencing the prognosis and treatment choices. Thus, quantitative MRI and PET imaging will play an increasingly important role in the management of patients in the coming years. Clinically, the use of multimodal imaging has increased with applications to differentiate BM from other brain tumors, assessing tumor responses to therapy, and management of treatment-induced late effects such as inflammation and radiation necrosis.^59^

In preclinical settings, quantitative MRI techniques have been used extensively in a variety of animal models. In particular, DCE-MRI and cellular MRI have revealed important information on single cell dissemination, early detection of BM, and the permeability of the BBB and the tumor vasculature. However, results from drug studies have to date shown little translational value,^4^ possibly due to inherent limitations when imaging animal models. For instance, reliable results on MRS may need voxel sizes of around 3.0 mm.^105^ Since almost all model systems to date are based on mice being injected with BM cells into the bloodstream, single tumor volumes of such sizes are usually not obtained. Thus, injections of BM cells directly into animal brains resulting in larger tumors would be easier to analyze with proton spectroscopy, leading to more well-founded outcomes and robust data.

For some of the MRI techniques (for instance DWI, ASL, and MRS), there seems to be a lack of standardization among laboratories. This may give rise to variability in the reported results from therapeutic studies, making translation to the clinic harder. An intriguing idea would be to initiate multi-center, preclinical studies to synchronize model systems, and MRI protocols, to obtain stronger conclusions related to therapeutic efficacies.

Cellular MRI has to date found little translation to the clinic. The use of CA targeted towards tumor cells^106^ and tumor vasculature^10^ could represent a promising strategy for earlier tumor detection and treatment in the future. Also, the results from the ongoing Phase II trial using feromyxotyl-based CA and gadolinium-based CA in perfusion imaging of NSCLC BM patients could be important for future clinical management of BM patients.

In the clinic, the value of using [^18^F]FDG in aiding clinical management of BM is questionable, due to its high uptake in the normal brain.^83^ The amino acid PET tracers [^11^C]MET, [^18^F]FET, [^18^F]FDOPA, and [^11^C]AMT represent more promising alternatives to differentiate between residual or recurrent BM, radiation necrosis, treatment-related changes, and pseudoprogression. Other radiotracers have also been studied to some extent, for tumor detection, and distinction between BM and other brain malignancies.

Preclinically, only PET studies using [^18^F]FDG and [18F]FLT have been reported to date. Relatively few BM research laboratories world-wide have the necessary technology and expertise available for PET imaging of BM model systems. Also, preclinical PET has a relatively low resolution compared to MRI. However, the excellent sensitivity of PET should encourage the research community to develop animal models and imaging protocols for the assessment of tumor development and treatment responses. Furthermore, PET could be used to study uptake, concentration and distribution of labelled drugs in BM. In this context, the results from preclinical studies may aid in tailoring personalized patient treatments. This has already been demonstrated in a recent preclinical study of HER2^+^ BMs from breast cancer, where [^89^Zr]trastuzumab was used in PET imaging. Specific uptake of the antibody in the brain due to BTB leakage was shown.^107^

Textural feature analysis (radiomics) of inconclusive lesions visualized on PET and MR images is entering the clinic, which may be able to differentiate radiation related injuries from recurrent BM.^108^ Further studies using larger populations, harmonization of analytical methods including machine learning among centers and inclusion of histopathological data would be warranted, to bring the technology into the clinic as a standard diagnostic tool. In this context, future preclinical studies using BM models should add valuable information that can be used for clinical translation.

Combined MR/PET systems are now being used in the clinic as well as in preclinical research centers around the world. Future use of MR/PET will advance current BM research, by combining the strengths and minimizing the limitations of each individual modality. Also, increased standardization of imaging protocols and study design among preclinical centers would likely bring the knowledge frontier forward, thereby increasing the translational value.

## Supplementary Material

vdab151_suppl_Supplementary_Table_S1

vdab151_suppl_Supplementary_Materials
