# Supplementary material for: Current landscape and future perspectives in preclinical MR and PET imaging of brain metastasis
Source: Neurooncol Adv. 2021 Oct 14;3(1):vdab151. doi: 10.1093/noajnl/vdab151 (PMC8704384; doi:10.1093/noajnl/vdab151)
Supplement: vdab151_suppl_Supplementary_Table_S1 [file vdab151_suppl_supplementary_table_s1.docx]

**Supplementary Table 1.** Typical spatial resolution of MRI techniques used in preclinical imaging of brain metastasis.

| **MRI Technique** | **Typical spatial resolution** | **Preclinical References** |
| --- | --- | --- |
| Anatomical MRI | 0.078 mm | 8 |
| bSSFP imaging | 0.1 mm | 23 |
| DSC-MRI | 0.31 mm | 29 |
| DCE-MRI | 0.078 mm | 37 |
| ASL | 0.5 mm | 41 |
| DWI | 0.156 mm | 44 |
| DTI | Not used preclinically | None |
| Cellular MRI | 0.078 mm | 8, 55 |
| CEST imaging | 0.25 mm | 63 |
| ^1^H-MRS | 3 mm | 44, 67 |
| ^31^P-MRS | Not used preclinically | None |
| BOLD | 0.31 mm | 71 |

**Abbreviations:** bSSFP balanced steady-state free precession, DSC dynamic susceptibility contrast, DCE dynamic contrast enhanced, ASL arterial spin labelling, DWI diffusion weighted imaging, DTI diffusion tensor imaging, CEST chemical exchange saturation transfer, ^1^H-MRS proton magnetic resonance spectroscopy, ^31^P-MRS phosphorus magnetic resonance spectroscopy, fMRI functional magnetic resonance imaging, BOLD blood oxygenation level-dependent imaging.
