# Supplementary material for: Current landscape and future perspectives in preclinical MR and PET imaging of brain metastasis
Source: Neurooncol Adv. 2021 Oct 14;3(1):vdab151. doi: 10.1093/noajnl/vdab151 (PMC8704384; doi:10.1093/noajnl/vdab151)
Supplement: vdab151_suppl_Supplementary_Materials [file vdab151_suppl_supplementary_materials.pdf]

Supplementary information

Literature search

A PubMed search for articles published up to October 13th 2020 was performed using the following search terms in combinations: “Magnetic resonance imaging”, “MR”, “MRI”, “positron emission tomography”, “PET”, “clinical”, “preclinical”, “animal models”, “mice”, “rats”, “brain metastases” and “brain metastasis”. Further, the reference lists of selected studies and authors were searched to identify additional relevant articles. All studies recognized were assessed for relevance by checking title, abstract and full text. Searches were conducted by three of the authors (SNA, HE, FT).
